# Mode of Discovering the Proper Capsule of the Crystalline Lens

**Published:** 1815-12

**Authors:** 


*For the London Medical and Physical Journal.*

### *Mode of Discovering the proper Capsule of the Crystalline Lens;* by N. L.

THAT differences of opinion should exist on various points of physiology and pathology, is not to be wondered at; and that discoveries should be made in animal structures at such a late period as the nineteenth century, is no more than one might expect; but that different anatomists, with equal opportunities for information, should give different descriptions of similar parts, is only to be accounted for from the prejudices, inattentions, or misdirected modes of dissection adopted by individuals.

One instance of such difference of description of the same structure, by modern authors and teachers, every anatomist must recollect, when I ask,—Has the crystalline lens a proper capsule?

Of its existence, any one may easily be satisfied, at least in the eyes of oxen, sheep, &c. by the following mode of dissection. Having separated the crystalline, and vitrious humours included in the common capsule, from the rest of the eye, make a small cut through the capsules on the fore part of the crystalline humour; then, by taking hold of the cut edges, each with a pair of forceps, gradually tear the capsule to the edge of the lens; then, having removed the lens, continue the rent, when it will be found to extend in two opposite directions, one through the vitrious tunic, as far as the outer side of the canal of Petit, and in another direction behind the situation of the lens, through its capsula propria, leaving the vitrious tunic behind it perfect. Observing this, I endeavoured to separate the capsula propria of the lens, entire, by cutting through the anterior lamina of the vitrious tunic, forming the canal, all round the edge of the lens; after which the capsula propria may easily be separated from that lamina of the vitrious tunic which is continued behind the lens; and you will thus easily obtain either humour apart from the other, and included in a perfect capsule. After repeated trials, I found the anterior lamina mina of the vitrious tunic, which commences at the outer edge of the canal, and is continued anterior to the capsula propria of the lens, was inseparable from it. The correct description, therefore, of this structure, is that given by Fyfe and others; whereas the conclusion of a tunica propria to the lens, though supported by many respectable anatomists, seems wrong. This discrepancy had caused me also to doubt its existence, before I adopted this mode of dissection.

*Cornwall; October* 2, 1815.

